# Scientific Publication Trend: Using Advanced Technologies in Cardiovascular Disease Research in the *Journal of Cardiovascular Development and Disease*

**DOI:** 10.3390/jcdd13020073

**Published:** 2026-02-02

**Authors:** Florin Anghel, Raluca-Oana Raianu, Zhonghua Sun

**Affiliations:** 1 Faculty of Medicine, Carol Davila University of Medicine and Pharmacy, 050474 Bucharest, Romania; florin.anghel@drd.umfcd.ro; 2 Emergency University Hospital of Bucharest, 050098 Bucharest, Romania; 3 Curtin Medical School, Curtin University, Perth 6845, Australia; 4 Emergency Clinical Hospital of Bucharest, 014461 Bucharest, Romania; raluca.raianu@gmail.com; 5 Curtin Medical Research Institute (Curtin MRI), Curtin University, Perth 6845, Australia

## 1. Introduction

Cardiovascular diseases (CVDs) remain the leading cause of mortality worldwide. As the life expectancy rises, the increasing burden of CVDs, marked by their growing incidence, impact on quality of life, and substantial economic costs, has intensified the demand for more precise management strategies [contribution 1].

For decades, clinical assessments have relied on standard imaging modalities, such as cardiac computed tomography (CT), magnetic resonance imaging (MRI), and echocardiography. These are common and non-invasive imaging examinations that are widely available and offer a high degree of diagnostic accuracy when compared to invasive angiographic examinations. Today, the diagnostic armamentarium is expanding to include artificial intelligence (AI), machine learning (ML), and deep learning (DL) algorithms, with increasing use in the routine diagnostic approach for CVDs. These technologies are shifting the paradigm from the simple observation and diagnosis of CVDs to predictive analytics, which improves the risk stratification, outcome prediction, and prevention of complex CVDs [1,2,3].

Simultaneously, imaging hardware has undergone a revolutionary change. Emerging technologies, like photon-counting computed tomography (PCCT), are now offering unprecedented spatial resolution and tissue characterization [4]. Furthermore, the utility of these CT acquisitions is being maximized through advanced visualization modalities. Advanced technologies such as Virtual Reality (VR), Augmented Reality (AR), and Mixed Reality (MR) are transcending traditional screen-based two-dimensional (2D) visualizations. These innovative tools offer immersive three-dimensional (3D) environments that enhance medical education and preoperative planning and improve patient health literacy [5,6].

Beyond diagnostics, these immersive technologies are reshaping surgical training. The integration of three-dimensional printing and VR simulations creates high-fidelity models for congenital and adult heart surgery, as well as other cardiovascular anomalies; 3D printing provides realistic and physical models of the cardiovascular anatomy and pathology, while VR offers a virtual environment that enables the user to explore cardiovascular diseases interactively with the digital data and even within real physical environments. These tools are crucial for the next generation of surgeons as they provide a safe and realistic environment to master complex procedures and life support protocols before entering the operating room [7,8].

This editorial aims to analyze the research trend of scientific publications in cardiovascular disease by utilizing advanced technologies over the last five years in an international open access journal, the *Journal of Cardiovascular Development and Disease* (JCDD). The rationale for conducting this analysis is to provide readers with an update on the research trends regarding the use of the latest technologies in CVD contexts.

A combination of search terms comprising AI, 3D printing, VR, AR, MR, and XR (extended reality which encompasses VR, AR, and MR) as well as PCCT were used to locate studies that utilize these advanced technologies in cardiovascular research in the JCDD over a period of five years (2021–2025). A total of 60 articles were included for analysis. The data extraction characteristics included study types (whether it is original research, a narrative review or systematic review, or case reports) and key findings.

We identified 60 articles that were published in the JCDD between 2021 and 2025. The majority of these studies were related to AI and DL research (n = 43, 71.7%), followed by the use of 3D printing (n = 11, 18.3%), VR/MR/XR (n = 9, 15%), and PCCT (n = 3, 5%). Six studies were extracted more than once due to reporting on multiple modalities, leading to a total of 66 analyzed reports. Within this analysis, more than half were original studies, the majority of which were AI-related. Narrative reviews represented a significant portion of the included literature, predominantly within the AI domain. There was only one systematic review (AI) and two case reports (covering AI and 3D printing). The three review articles regarding PCCT focused exclusively on technical and clinical applications in coronary artery disease. In the following sections, we provide a detailed analysis of the studies that examine the use of advanced technologies in CVDs.

## 2. Analysis of the Study Trend

### 2.1. Artificial Intelligence (AI): Advanced Diagnostic and Predictive Models

The AI domain represented the largest portion of the analyzed literature. The shift towards the automation of diagnostic processes and the interest in complex predictive analysis tools were identified as main implementation methods in the prevention, diagnosis, and therapy of multiple cardiovascular diseases.

Khanna et al. demonstrated that AI-based tissue characterization—using ML and DL—is effective for assessing vascular damage in COVID-19 patients across pulmonary, renal, and coronary vessels [contribution 2]. In the management of hypertension, Visco et al. noted that AI solutions improve the understanding of epigenetics and risk stratification, especially when combined with wearables to detect non-adherence to therapy [contribution 3].

**Figure 1:**
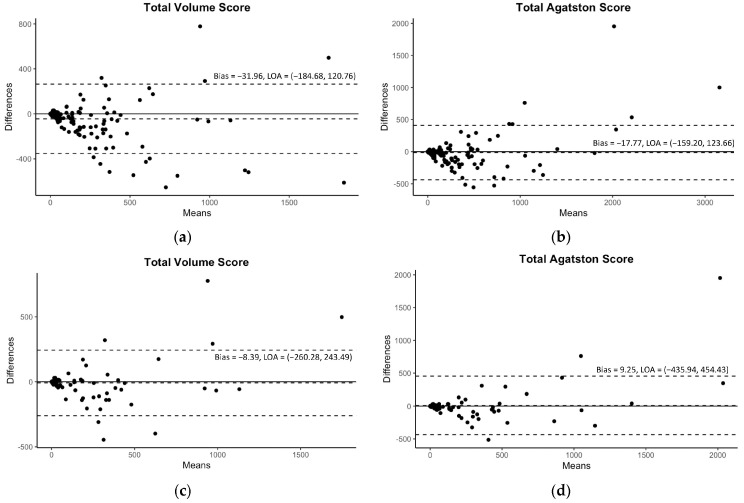
The Bland–Altman plots for the predicted volume scores and Agatston scores with 95% limits of agreement (LOAs). The plots of the internal validation are presented in (**a**,**b**) for the total volume score and Agatston score, respectively. The plots of the external validation are presented in (**c**,**d**) for the same scores. Reprinted with permission under open access protocols from Lee et al. [contribution 6].

Diagnostic precision is another pillar of current AI research. Manuel et al. found that an AI-driven ECG analysis matches or exceeds the results of experienced clinicians in identifying arrhythmias [contribution 4]. Furthermore, Sawchuk utilized AI to analyze the electronic medical records (EMRs) of 872 patients, finding that Support Vector Machines could predict stroke or transient ischemic attacks in patients with carotid stenosis with a high sensitivity [contribution 5]. For the risk stratification of coronary artery disease, Lee et al. confirmed that their developed DL-based automated model is highly accurate in extracting coronary artery calcium from contrast-enhanced coronary CT angiography with high fidelity without requiring extra radiation doses (Figure 1) [contribution 6].

### 2.2. Three-Dimensional Printing: From 3D-Printed Models and Surgical Simulators to Tissue Engineering

Three-dimensional printing is increasingly valuable in cardiovascular medicine, moving beyond anatomical modeling to bio-engineering and precise surgical planning; 3D-printed models have several functions: medical education, preoperative planning and simulations, hands-on training, procedure simulations, improved clinical communication, the optimization of CT protocols, and the treatment of cardiovascular diseases with bioprinted devices like stents, valves, and cardiac patches [contribution 1] [9,10,11]. Work on the use of 3D printing technology in cardiovascular disease represents the second most commonly reported studies in the JCDD according to our analysis. The key findings for 3D printing underscore its role in enhancing surgical precision and developing new personalized approaches. Banzhkina et al. reported on the successful 3D bioprinting of cardiac patches for heart repair, using auxetic designs to create adaptable mechanics that match the properties of the myocardium. This study demonstrated that a 0.2 mm thick patch exhibited the myocardium’s mechanical properties [contribution 7].

**Figure 2:**
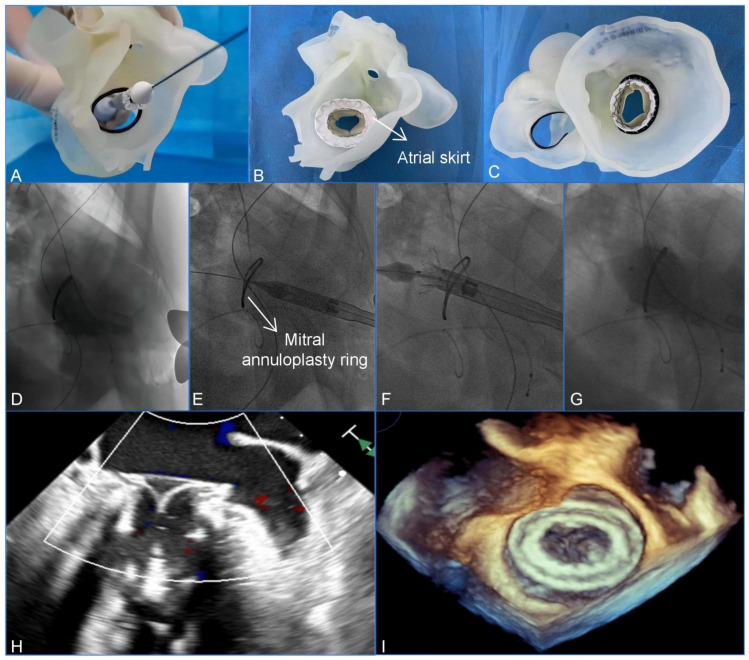
A preoperative assessment and simulation of the main procedural steps for a transcatheter mitral valve-in-ring replacement on patient-specific 3D-printed models. (**A**) The adjustment of the coaxiality and the release position. (**B**,**C**) The stent was fully unfolded and observed in the left atrial and the ventricular views. (**D**–**G**) The main steps of the procedure. (**D**) Fluoroscopy revealed severe mitral regurgitation. (**E**) The delivery system was advanced via the transapical approach. (**F**) The initial release of the stent. (**G**–**I**) After the stent was fully released, fluoroscopy and transesophageal echocardiography revealed that the bioprosthesis was in a stable position and functioning well without paravalvular leakage. Reprinted with permission under an open access protocol from Wang et al. [contribution 8].

Clinically, 3D printing is essential for assisting with the management of complex cardiac anatomies. Wang et al. utilized a patient-specific 3D-printed model to guide a transcatheter mitral valve-in-ring (TMViR) replacement, which assisted with valve sizing and anticipating intraoperative complications. This study confirms new clinical directions, such as endovascular navigation enabled through the use of 3D-printed models [contribution 8] (Figure 2).

The tangible benefit of this technology was quantified by Kolcz et al., who conducted a comparative cohort study on infants undergoing Major Aortopulmonary Collateral Artery (MAPCA) unifocalization [contribution 9]. They found that the intervention group that utilized 3D printing and VR had significantly shorter operative and cardiopulmonary bypass times compared to the control group. Also, cardiac tumors, very rare pathologies with an unpredictable anatomy, could benefit from this technology [12,13].

### 2.3. VR, AR, MR, and XR: Immersive Planning and Educational Tools

Immersive technologies such as VR, AR, and MR are proving to be powerful decision support and educational tools, and use of these technologies in CVD contexts represents the third most commonly documented research trend in the JCDD. Abjigitova et al. found that VR improved surgeons’ understanding of anatomy so significantly that it changed the surgical decision in 33% of aortic surgery cases [5]. Lee and Lau et al. corroborated this, stating that VR provides the most accurate evaluation of complex spatial cardiac structures, making it the superior presurgical tool for preoperative planning when compared to 3D printing models and original cardiac CT images [contribution 1] [9].

**Figure 3:**
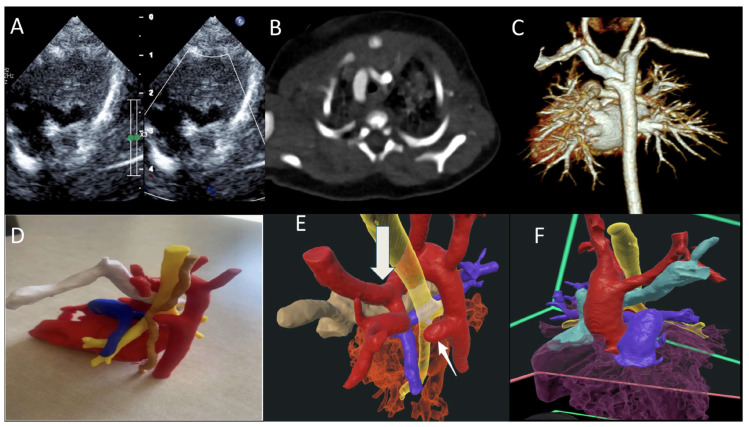
(**A**) TTE showing double aortic arch with dominant right arch arrow (DAA); (**B**,**C**) CT scan of the DAA with dominant right aortic arch, Kommerell diverticulum and atretic left arch; (**D**) 3D-printed model trachea (yellow), esophagus (brown), and retro-aortic innominate vein (white); and (**E**,**F**) VR showing DAA (red), trachea (yellow), and retro-aortic innominate vein (teal) ring, completed by the atretic left aortic arch and Kommerell diverticulum (arrow). TTE—transthoracic echocardiography; VR—virtual reality. Reprinted with permission under an open access protocol from Narasimhan et al. [contribution 10].

In medical training and patient care, the findings are equally transformative. Through a randomized controlled trial, Peek et al. demonstrated that VR training for cardiac pulmonary resuscitation (CPR) post-cardiac surgery led to a more accurate, albeit slower, performance among residents compared to conventional methods [7]. Regarding patient interaction, Godula et al. studied this impact in their randomized study through an analysis of 182 patients and compared VR-based education with the traditional physician-led education on hypertension, focusing on their impact on patient cooperation. The group that received the VR training had an advantage in knowledge acquisition compared to the group that received the traditional training. In terms of satisfaction, both methods received high ratings, suggesting that VR should serve as a complement to, rather than a replacement for, traditional in-person education, as human connection remains an essential element [6]. It has been reported that while 3D printing is valuable, extended reality offers the advantage of interactive 3D representations without the high production costs and time requirements associated with physical models [14]. A more recent study by Narasimhan and colleagues further validated the clinical value of VR for understanding CHD conditions. Twenty-six clinicians completed their survey: 100% considered VR as a valuable diagnostic tool, 81% believed that VR could enhance diagnostic accuracy and surgical planning, 50% highlighted its educational benefits, and all recommended the use of VR to their colleagues (Figure 3) [contribution 10].

### 2.4. Photon-Counting Computed Tomography (PCCT): The New Imaging Frontier

**Figure 4:**
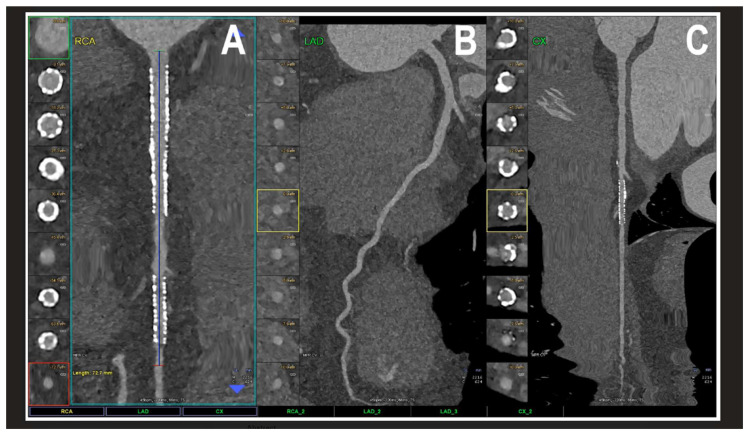
A cardiac PCCT visualization of the coronary stents and stented lumen. There are two stents at the level of the proximal and middle RCA (**A**) and one stent on the marginal branch of the left LCx (**C**); the LAD (**B**) is normal without any detectable atherosclerotic disease. All stents are perfectly visualized in their inner struts and also in their inner lumen, which is difficult to visualize with standard cardiac CT. PCCT—photon counting CT, LAD—left anterior descending, LCx—left circumflex, and RCA—right coronary artery. Reprinted with permission under open access guidelines from Cademartiri et al. [15].

The results regarding PCCT identify it as a revolutionary step in cardiovascular diagnostics. PCCT directly converts X-ray photons into electrical signals, offering a spatial resolution of 0.2 mm^2^, which is superior to traditional coronary CT angiography [4,15,16,17,18,19] [contribution 11 and contribution 12]. This technical advantage significantly reduces “calcium blooming,” which in turn minimizes the overestimation of coronary stenosis—a common pitfall in conventional CT. Furthermore, PCCT excels in the visualization of coronary plaques, patent lumens, and in-stent restenosis, offering a superior accuracy in quantifying luminal stenosis across all plaque types and characterizing the plaque composition, including its biological activity (Figure 4) [15,16,17,18,19].

Beyond coronary assessments, this technology enables the precise quantification of the myocardial extracellular volume and the accurate delineation of myocardial scars, which are essential for evaluating perfusion defects. While PCCT is a relatively new technology, its improved diagnostic efficacy is already reducing the necessity for invasive coronary angiography referrals, and future research is expected to uncover an even broader spectrum of clinical applications as the technology becomes more widely implemented. Despite only a few studies published in the JCDD, it is expected that more research will be available on the use of PCCT for CVDs in the near future—when the availability of the technology expands and it is used in many clinical sites—as well as with the increasing experience expected in this research field.

### 2.5. Clinical Relevance of Left Atrial Strain

The clinical utility of Left Atrial Strain (LAS) has emerged as a cornerstone of modern functional echocardiography, offering a highly sensitive and non-invasive means of assessing atrial mechanical performance and stiffness. Unlike traditional volumetric measurements, such as the Left Atrial Volume Index (LAVI), LAS, and particularly reservoir strain (LASr), can detect subclinical atrial dysfunction before structural remodeling becomes evident. Research published in the JCDD emphasizes its powerful predictive value in identifying the risk of atrial fibrillation (AF) recurrence following catheter ablation and its role as an independent marker for stroke risk. Furthermore, LAS is increasingly being integrated into the diagnostic framework for Heart Failure with preserved Ejection Fraction (HFpEF), where it helps unmask impaired atrial compliance and elevated filling pressures. The ongoing shift toward the AI-driven automation for LAS quantification further enhances its clinical relevance by reducing inter-observer variability and facilitating its transition into routine cardiovascular practice [20,21,22].

The transition of advanced technologies from research tools to clinical practice is governed by evidence-based guidelines from major societies such as the ESC and AHA/ACC. While traditional imaging remains the cornerstone of diagnosis, the clinical status of newer modalities like AI and 3D printing is evolving. Currently, standard echocardiography, magnetic resonance (MR), and CT for routine assessments are considered Class I recommendations. In contrast, emerging technologies like PCCT and specific AI applications are generally categorized as IIb, indicating that while evidence supports their usefulness, they are often reserved for complex cases or specialized centers.

While formal, universal guidelines are lacking, 3D printing in congenital heart disease (CHD) is used for planning complex surgeries, surgical training/simulation, and improving patient/parent education, especially for unique anatomies where standard methods fall short, with expert consensus suggesting its use for complex cases, like double outlet right ventricles (DORVs), to support decision-making, though more evidence on outcomes is needed. Key considerations include ensuring high-quality imaging (CT/MRI/echo) and balancing model quality with printing time, particularly in urgent situations [23]. AI is now integrated into several major international cardiology guidelines, though primarily as a screening and supportive tool rather than a standalone diagnostic for arrhythmias. Guidelines currently distinguish between AI as a tool for detection (e.g., smartwatches for atrial fibrillation) and AI as a predictor (e.g., identifying risk of future events from a normal ECG).

The 2020 ESC AF management guideline states that a 30 s single-lead electrocardiogram (sl-ECG) can be used to diagnose atrial fibrillation but advises caution as most validation studies use small, carefully selected cohorts and are prone to bias. Patients with frequent ectopy, other atrial arrhythmias, and cardiac implantable electronic devices, which are important sources of false-positive detection, are noticeably absent from many studies [24].

While PCCT technology (such as the NAEOTOM Alpha) has been FDA-cleared and in clinical use since 2021, its mention in formal guidelines typically highlights its role in addressing the limitations of conventional energy-integrating detectors (EIDs), such as heavy calcification and stent imaging. Groups like the Society of Cardiovascular Computed Tomography (SCCT) are increasingly incorporating PCCT data into expert consensus documents, particularly for ultra-high-resolution coronary assessments [25]. Table 1 is a summary of recommendations with regard to the use of these advanced technologies.

## 3. Summary

This editorial provides a summary of the research trend of using the latest technologies in the diagnostic assessment of cardiovascular diseases in the *Journal of Cardiovascular Development and Disease* over a period five years. We identified 60 articles that examine the use of AI, 3D printing, VR/AR/MR, and PCCT in cardiovascular diseases and observed that use of AI represents the primary research focus within the JCDD, accounting for over 70% of analyzed publications. This phenomenon is within expectations given the rapid development of AI algorithms in the medical domain, especially in the cardiovascular disease field.

Three-dimensional printing has demonstrated immense promise in enabling personalized surgical approaches, particularly for complex congenital heart disease and high-risk transcatheter procedures. With more printing materials available to replicate human anatomical structures and properties, and printing costs being reduced, it is expected that further evidence will be available to prove the value of using 3D-printed personalized models in cardiovascular diseases.

Immersive 3D visualization technology (VR, AR, MR, and XR) has transitioned from a novel concept to a high-value tool for preoperative decision-making and medical education. Although most of the current studies mainly rely on the use of VR, there is an increasing trend of using AR and MR as the technology becomes mature. Research has also proven the clinical value of VR-related 3D visualization tools in various cardiovascular diseases, from medical education to surgical planning and simulations of surgery procedures. While still in its early stages of clinical implementation, photon-counting computed tomography represents a revolutionary leap in non-invasive diagnostics due to its many advantages over current CT scanners. The collective integration of AI, 3D printing, and immersive visualization is moving cardiovascular medicine toward a highly personalized framework.

## Figures and Tables

**Table 1:** Summary of current clinical recommendation status for advanced cardiovascular technologies.

Technology	Clinical Application	Recommendation Class
Standard Imaging (Echo, MR, CT)	Routine assessment and cornerstone of diagnosis	Class I
Artificial Intelligence (AI)	Arrhythmia screening, detection (e.g., AF), and predictive modeling	Class IIb
3D Printing	Preoperative planning for complex CHD, surgical training, and education	Class IIb
Photon-Counting CT (PCCT)	High-resolution coronary assessment, calcification, and stent imaging with improved accuracy	Class IIb

## Abbreviations

The following abbreviations are used in this manuscript:

2D	Two-dimensional
3D	Three-dimensional
CABG	Coronary artery bypass grafting
CT	Computed tomography
CVDs	Cardiovascular diseases
AI	Artificial intelligence
AR	Augmented reality
DAA	Double aortic arch
DL	Deep learning
EID	Energy integrating detector
JCDD	*Journal of Cardiovascular Development and Diagnosis*
LAD	Left anterior descending
LCx	Left circumflex
MAPCAs	Major aortopulmonary collateral arteries
MRI	Magnetic resonance imaging
ML	Machine learning
MR	Mixed reality
PCCT	Photon-counting computed tomography
RCA	Right coronary artery
TMViR	Transcatheter mitral valve-in-ring
TTE	Transthoracic echocardiography
VR	Virtual reality
XR	Extended reality

## References

[1] Sadr H., Salari A., Ashoobi M.T., Nazari M. (2024). Cardiovascular disease diagnosis: A holistic approach using the integration of machine learning and deep learning models. Eur. J. Med. Res..

[2] Zhang X.D., Tian T., Liao L.Z., Dong Y.H., Zhou H.J., Zhang S.Z., Chen W.Y., Du Z.M., Wang Y.Q., Liao X.X. (2022). Deep phenotyping and prediction of long-term cardiovascular disease: Optimized by machine learning. Can. J. Cardiol..

[3] Wu Z., Guo C. (2025). Deep learning and electrocardiography: Systematic review of current techniques in cardiovascular disease diagnosis and management. Biomed. Eng. Online.

[4] Sharma A., Cerdas M.G., Reza-Soltani S., Rustagi V., Guntipalli M., Rojas Torres D.S., Bhandari M., Kandel S., Teja Rayaprolu D., Hussain M. (2024). A review of photon-counting computed tomography (PCCT) in the diagnosis of cardiovascular diseases. Cureus.

[5] Jung C., Wolff G., Wernly B., Bruno R.R., Franz M., Schulze P.C., Silva J.A., Silva J.R., Bhatt D.L., Kelm M. (2022). Virtual and augmented reality in cardiovascular care: State-of-the-art and future perspectives. JACC Cardiovasc. Imaging.

[6] Goo H.W., Park S.J., Yoo S.J. (2020). Advanced medical use of three-dimensional imaging in congenital heart disease: Augmented reality, mixed reality, virtual reality and three-dimensional printing. Korean J. Radiol..

[7] Bartelli F., Singh A., Lam C.Z., Seatle H., Peel B., Yoo S.J., Daskalo C., Valverde I. (2025). Advancements in 3D modelling technologies for congenital heart disease: Integrating 3D printing, virtual reality and holograms. Pediatr. Radiol..

[8] Stepanenko A., Perez L.M., Ferre J.C., Falcon C.Y., Perez de la Sota E., San Roman J.A., Redondo Diegues A., Baladron C. (2023). 3D virtual modelling, 3D printing and extended reality for planning of implant procedure of short-term and long-term mechanical circulatory support devices and heart transplantation. Front. Cardiovasc. Med..

[9] Lau I., Gupta A., Ihdayhid A., Sun Z. (2022). Clinical applications of mixed reality and 3D printing in congenital heart disease. Biomolecules.

[10] Sun Z. (2023). Patient-specific 3D-printed models in pediatric congenital heart disease. Children.

[11] Sun Z., Zhao J., Leung E., Flandes-Iparranguirre M., Vernon M., Silberstein J., De-Juan-Pardo E.M., Jansen S. (2023). Three-dimensional bioprinting in cardiovascular disease: Cirrent status and future directions. Biomolecules.

[12] Liddy S., McQuade C., Walsh K.P., Loo B., Buckley O. (2019). The assessment of cardiac masses by cardiac CT and CMR including pre-op 3D reconstruction and planning. Curr. Cardiol. Rep..

[13] Hajj J., Harb S.C., Klatte R.S., Ondrejka S.L., Valent J., Rosinski B., Roselli E.E., Tong M.Z.Y. (2025). Primary cardiac lymphoma masquerading as a pericardial mass: Imaging, 3D printing and surgical management. JACC Case Rep..

[14] Raimondi F., Vida V., Godard C., Bertelli F., Reffo E., Boddaert N., El Beheiry M., Masson J.B. (2021). Fast-track virtual reality for cardiac imaging in congenital heart disease. J. Card. Surg..

[15] Cademartiri F., Meloni A., Pistoia L., Degiorgi G., Clemente A., Gori C.D., Positano V., Celi S., Berti S., Emdin M. (2023). Dual-Source Photon-Counting Computed Tomography—Part I: Clinical Overview of Cardiac CT and Coronary CT Angiography Applications. J. Clin. Med..

[16] Si-Mohamed S.A., Boccalini S., Lacombe H., Diaw A., Varasteh M., Rodesch P.-A., Dessouky R., Villien M., Tatard-Leitman V., Bochaton T. (2022). Coronary CT angiography with photon-counting CT: First-in-human results. Radiology.

[17] Flohr T., Schmidt B., Ulzheimer S., Alkadhi H. (2023). Cardiac imaging with photon counting CT. Br. J. Radiol..

[18] Allmendinger T., Nowak T., Flohr T., Klotz E., Hagenauer J., Alkadhi H., Schmidt B. (2022). Photon-Counting Detector CT-Based Vascular Calcium Removal Algorithm: Assessment Using a Cardiac Motion Phantom. Investig. Radiol..

[19] Boccalini S., Si-Mohamed S.A., Lacombe H., Diaw A., Varasteh M., Rodesch P.A., Villien M., Sigovan M., Dessouky R., Coulon P. (2022). First In-Human Results of Computed Tomography Angiography for Coronary Stent Assessment with a Spectral Photon Counting Computed Tomography. Investig. Radiol..

[20] Monte I.P., Bottari V., Licciardi S., Trovato G., Russo M.S., Campisi M., Di Stefano G., Sclafani S., Galassi A.R., Tamburino C. (2023). Left Atrial Strain Imaging by Speckle Tracking Echocardiography: The Supportive Diagnostic Value in Cardiac Amyloidosis and Hypertrophic Cardiomyopathy. J. Cardiovasc. Dev. Dis..

[21] Barilli M., Al-Mohaissen M.A., Di Mauro M., Giammarco G.D., Pepi M., Gallina S. (2024). Potential Role of Left Atrial Strain to Predict Atrial Fibrillation Recurrence after Catheter Ablation Therapy: A Clinical and Systematic Review. J. Cardiovasc. Dev. Dis..

[22] Gan G.C., Shanker A., Sivasampu S., Bhaskaran A., Kizana E., Thomas L. (2022). Left Atrial Strain and the Risk of Stroke in Patients with Atrial Fibrillation. J. Cardiovasc. Dev. Dis..

[23] Hussein N., Valverde I., Honjo O., Balaji A., Barron D.J., Yoo S.J. (2025). Expanding the use of 3D printing in congenital heart surgery. Transl. Pediatr..

[24] Van Gelder I.C., Rienstra M., Bunting K.V., Casado-Arroyo R., Caso V., Crijns H.J.G.M., De Potter T.J.R., Dwight J., Guasti L., Hanke T. (2024). 2024 ESC Guidelines for the management of atrial fibrillation developed in collaboration with the European Association for Cardio-Thoracic Surgery (EACTS). Eur. Heart J..

[25] Cademartiri F., Maffei E., Cau R., Positano V., De Gori C., Celi S., Saba L., Bossone E., Meloni A. (2025). Current and Future Applications of Photon-Counting Computed Tomography in Cardiovascular Medicine. Heart.

